# Bilateral petrous apex cephaloceles: Is surgical intervention indicated?

**DOI:** 10.1016/j.ijscr.2020.06.021

**Published:** 2020-06-11

**Authors:** Ali Alkhaibary, Fahd Musawnaq, Makki Almuntashri, Abdulaziz Alarifi

**Affiliations:** a College of Medicine, King Saud bin Abdulaziz University for Health Sciences, Riyadh, Saudi Arabia; b King Abdullah International Medical Research Center, Riyadh, Saudi Arabia; c Division of Neurosurgery, Department of Surgery, King Abdulaziz Medical City, Ministry of National Guard - Health Affairs, Riyadh, Saudi Arabia; d Medical Imaging Department, King Abdulaziz Medical City, Riyadh, Saudi Arabia

**Keywords:** PAC, petrous apex cephalocele, CSF, cerebrospinal fluid, Bilateral, Case report, Cephalocele, Headache, Petrous, Sella

## Abstract

**Introdcution:**

Petrous apex cephaloceles are characterized by herniation of Meckel’s cave into the petrous apex. An extensive review of the literature reveals 20 cases of bilateral petrous apex cephaloceles. This article reports an additional case of bilateral petrous apex cephaloceles and reviews the pertinent literature.

**Presentation of case:**

A 64-year-old female was referred from a primary care clinic due to longstanding headache. A non-enhanced CT scan of the brain revealed osteolytic bony lesions at the petrous apices and an empty sella. A brain MRI with contrast showed CSF-containing lesions in the petrous apices, communicating with Meckel’s cave bilaterally. The patient was managed conservatively and is currently followed up in the neurosurgery clinic.

**Discussion:**

While the exact etiology remains uncertain, petrous apex cephaloceles are postulated to originate from sustained, chronic elevation of intracranial pressure. On MRI, petrous apex cephaloceles display signal intensities resembling CSF throughout all sequences. They demonstrate well-defined margins continuous with Meckel’s cave. CT scans allow further characterization, i.e. invasive erosions, of the osseous structures in patients with petrous apex cephaloceles.

**Conclusion:**

A thorough understanding of the petrous apex anatomy and its pathological lesions is paramount. A brain MRI remains the diagnostic imaging of choice to characterize petrous apex cephaloceles.

## Highlights

• Petrous apex cephaloceles (PAC) are rare lesions with typical imaging features.
• Most PACs are unilateral, incidental findings known as “leave-me-alone” lesions.
• Bilateral PACs are commonly associated with empty sella syndrome.

## 1 Introduction

The petrous apex is a pyramidal-shaped structure located within the medial portion of the temporal bone that may harbor several anatomical and pathological lesions [1]. Considering the intricate anatomical location of the petrous apex and the inability to directly examine such lesions therein, a radiographic assessment to delineate petrous apex lesions is of utmost importance. Petrous apex lesions are categorized into either; lesions mandating a surgical intervention or non-operative lesions that are discovered incidentally [2].

Petrous apex cephaloceles (PAC) are rare lesions that are characterized by cystic appearance, on radiological imaging, and extension of the posterolateral aspect of Meckel’s cave into the superomedial portion of the petrous apex [1,3]. Petrous apex cephaloceles are usually asymptomatic lesions that can be unilateral or bilateral. The differential diagnosis of petrous apex cephaloceles is broad. However, it includes cholesterol granuloma, petrous apex effusions, cholesteatoma, apical petrositis, and mucoceles [3].

Considering the rarity of bilateral petrous apex cephaloceles, this article discusses the clinical presentation and radiological findings of a 64-year-old female diagnosed with bilateral petrous apex cephaloceles.

## 2 Case description

A 64-year-old female, known to have diabetes and hypertension, was referred from a primary care clinic due to chronic headache. Further history revealed that the patient had chronic allergic sinusitis and laryngitis.

Physical examination revealed that the patient was alert and oriented to time, place, and person with a GCS of 15/15. There were no cranial nerves deficits. The muscle power was grade 5/5 in all myotomes. Sensation was intact to light touch, pinprick, and vibration. Deep tendon reflexes (DTR) were grade +2 throughout. Fundoscopic examination revealed no papilledema. The pupils were 3 mm reactive to both light and accommodation with full extraocular movements. The patient had no diplopia or ptosis. The visual acuity of the patient was intact. Visual field confrontation revealed an unremarkable temporal and nasal vision.

**Fig. 1:**
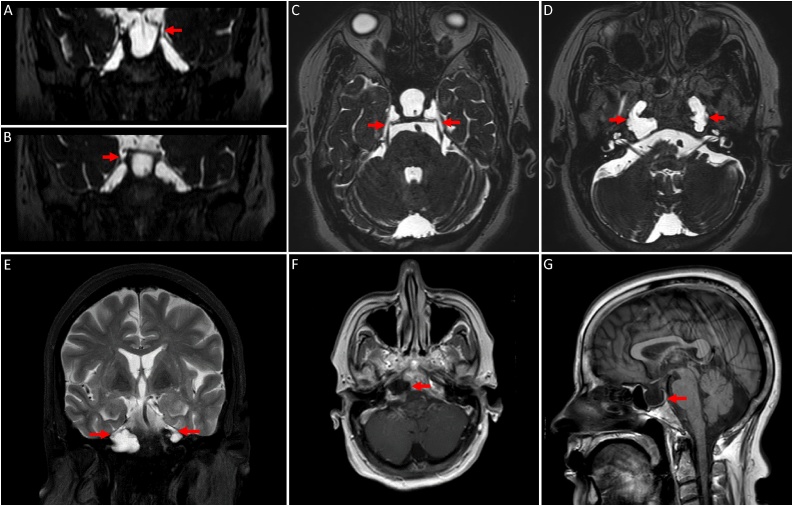
MRI of the brain; **(A-D)** Coronal and axial steady state sequences / FIESTA. **(E)** Coronal T2 weighted images / T2WI with fat suppression. **(F)** Axial T1WI post contrast. **(G)** Sagittal T1WI. **(A-F)** The images demonstrate bilateral, lytic, expansile, lobular lesions extending from Meckel’s caves into the petrous apices. These lesions follow CSF signal on all sequences with no enhancement. **(G)** An associated empty sella is noted.

**Fig. 2:**
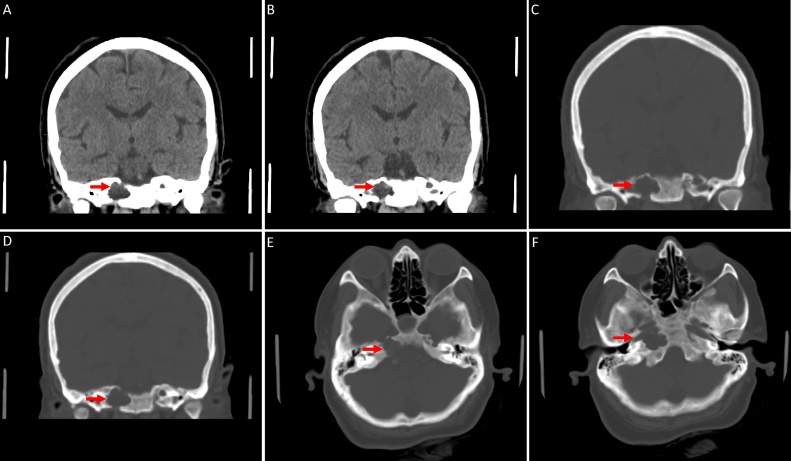
Nonenhanced CT brain; **(A-B)** Coronal brain window. **(C-F)** Coronal and axial bone window. **(A-F)** There are bilateral, well-defined, lytic, expansile, fluid-containing lesions in the petrous apices with bone dehiscence of the inner cortex of the petrous bone.

Neuroimaging studies were performed to investigate the possible causes of her headache. The brain MRI showed CSF-containing lesions with no enhancement in the petrous apices bilaterally, communicating with Meckel’s cave (Fig. 1). The non-enhanced CT scan of the brain revealed bilateral osteolytic bony lesions in the petrous apices with fluid density (Fig. 2).

Giving the benign nature of the lesion, neurosurgical intervention was not indicated. The patient is currently followed up in the neurosurgery clinic.

## 3 Discussion

Most petrous apex cephaloceles are unilateral, incidental findings that are referred to as “leave-me-alone” lesions [2]. Bilateral petrous apex cephaloceles, on the contrary, are extremely rare with only few cases reported in the literature. To our knowledge, a review of the literature revealed 20 cases of bilateral petrous apex cephaloceles. Table 1 outlines the clinical presentation, management, and the relation to empty sella syndrome of all reported cases in the literature.

**Table 1:** Summary of all the reported cases of bilateral petrous apex cephaloceles in the literature.

No.	Author, Year	Age/Sex	Presentation	Management	Empty Sella
**1**	Jakkani R [8], 2012	39/M	Headache	NA	**+**
**2**	Hatipoglu HG [6], 2010	47/F	Headache	NA	**+**
**3**	Hatipoglu HG [6], 2010	60/F	Headache	NA	**+**
**4**	Hatipoglu HG [6], 2010	46/F	Headache	NA	–
**5**	Hatipoglu HG [6], 2010	41/F	Diplopia	NA	**+**
**6**	O’connell BP [5], 2016	56/F	Tinnitus	NA	**+**
**7**	Moore KR [2], 2001	48/F	Left III & V palsy	Surgical	–
**8**	Moore KR [2], 2001	82/F	Bilateral SNHL	Conservative	–
**9**	Moore KR [2], 2001	66/F	Head whooshing sensation	Conservative	–
**10**	Canan A [9], 2017	61/F	Tinnitus	Conservative	–
**11**	Alorainy IA [3], 2007	25–60/F	NA	Conservative	**+**
**12**	Alorainy IA [3], 2007	25–60/F	NA	Conservative	**+**
**13**	Alorainy IA [3], 2007	25–60/F	NA	Conservative	**+**
**14**	Alorainy IA [3], 2007	25–60/F	NA	Conservative	**+**
**15**	Stark TA [10], 2009	58/M	NA	Conservative	–
**16**	Jeong BS [11], 2011	42/F	Tinnitus & hearing impairment	Conservative	**+**
**17**	Jeong BS [11], 2011	63/F	Headache & leg weakness	Conservative	**+**
**18**	Hervey-Jumper SL [12], 2010	14/M	Bacterial meningitis	Surgical	–
**19**	Kulkarni A [13], 2018	70/F	Headache	Conservative	–
**20**	Kulkarni A [13], 2018	63/M	Headache & vertigo	Conservative	–
**21**	Present Case, 2020	64/F	Headache	Conservative	**+**

**M:** Male; **F:** Female; **SNHL:** Sensorineural Hearing Loss; **NA:** Not Available; **(+):** Present; **(–):** Not present.

Around 80% (n = 16) of the reported cases were diagnosed in the female population. The youngest patient was diagnosed at the age of 14, whereas the oldest patient was diagnosed at the age of 63. Most patients (n = 7; 35%) presented with recurrent headaches. Tinnitus was the second most frequent complaint (n = 3; 15%). Most patients were managed conservatively, and only two patients underwent surgical intervention.

Approximately half of the reported cases (n = 11; 55%) were associated with empty sella syndrome. This makes the present case the twelfth of its kind depicting simultaneous bilateral petrous apex cephaloceles and empty sella syndrome.

To date, the exact etiology of petrous apex cephaloceles remains uncertain. However, the most widely accepted theory explaining the development of petrous apex cephaloceles is altered CSF dynamics, leading to increased intracranial pressure in a congenitally thin petrous apex bone [3,4]. In turn, this leads to a herniation of the meninges and CSF through the weak points within the skull from the posterolateral portions of Meckel’s cave. This might explain the co-occurrence of petrous apex cephaloceles with empty sella syndrome [3,4].

Although petrous apex cephaloceles are usually asymptomatic lesions, they are considered as possible causes of CSF otorrhea, rhinorrhea, vertigo, trigeminal neuralgia, and meningitis [5]. If petrous apex cephaloceles are symptomatic, surgical intervention might be considered. However, a thorough clinical and radiological assessment should be performed to exclude possible causes contributing to symptomatic petrous apex cephaloceles [2,6].

It is imperative to differentiate petrous apex cephaloceles from other lesions involving the petrous apex. A combination of CT scan and MRI, including diffusion weighted and post gadolinium imaging, can be considered to delineate such lesions [6].

On MRI, petrous apex cephaloceles display signal intensities resembling CSF throughout all sequences. They are homogenously hypointense on T1-weighted and hyperintense on T2-weighted MRI. They do not demonstrate restriction on diffusion weighted images nor enhancement following contrast injection. They demonstrate well-defined margins that are continuous with Meckel’s cave [5]. CT scans allow further characterization, i.e invasive erosions, of the petrous apex osseous structures [7]. In the present case, bilateral osteolytic changes of the petrous apices were noted, suggesting a long-standing disease process.

## 4 Conclusion

Bilateral petrous apex cephaloceles are rare lesions with typical imaging features. Understanding the anatomy of the petrous apex and its pathological lesions help in characterizing and establishing the diagnosis. A brain MRI remains the diagnostic imaging of choice. Most petrous apex cephaloceles are discovered incidentally in patients presenting with chronic headaches. Patients with bilateral petrous apex cephaloceles can be managed conservatively with regular clinical and radiological follow-up in the neurosurgery clinic.

## Declaration of Competing Interest

The authors declare that they no conflict of interest.

## Sources of funding

This research did not receive any specific grant from funding agencies in the public, commercial, or not-for-profit sectors.

## Ethical approval

Case reports do not require ethical approval if the images and clinical data are anonymized.

## Consent

Written informed consent was obtained from the patient for publication of this case report and accompanying images.

## Author contribution

**Ali Alkhaibary:** Conceptualization, Investigations, Writing - Original Draft, Writing - Review and Editing. **Fahd Musawnaq:** Conceptualization, Investigations, Writing - Original Draft, Approval of final manuscript. **Makki Almuntashri:** Radiological description, Writing - Original Draft, Approval of final manuscript**. Abdulaziz Alarifi:** Conceptualization, Supervision, Approval of final manuscript.

## Registration of research studies

Not applicable.

## Guarantor

Ali Alkhaibary.

## Provenance and peer review

Not commissioned, externally peer-reviewed.

## Ethical consideration

Radiographic images were anonymized to maintain the patient’s privacy. Informed consent was taken from the patient prior to collecting her data. This case was reported in accordance with the SCARE criteria [14].
